# Biological Therapy with Complementary and Alternative Medicine in Innocuous Integrative Oncology: A Case of Cervical Cancer

**DOI:** 10.3390/pharmaceutics13050626

**Published:** 2021-04-28

**Authors:** Elvin Peter Chizenga, Heidi Abrahamse

**Affiliations:** Laser Research Centre, University of Johannesburg, Johannesburg 2028, South Africa; elvinc@uj.ac.za

**Keywords:** biological therapy, cervical cancer, human papillomavirus (HPV), cancer hallmarks, integrative medicine, nutraceuticals, oncoproteins, tumor-associated antigens (TAAs)

## Abstract

Good medicine is based on good science, inquiry driven and open to new paradigms. For a complex disease such as cancer, a complex treatment regime that is well structured and multifactorial is indispensable. In the present day, Complementary and Alternative Medicine (CAM) therapies are being used frequently for cancer, alongside modern biological therapies and allopathic medicine, in what is called integrative oncology. In all conscience, the use of natural, less invasive interventions whenever possible is ideal. However, a comprehensive understanding of not only the etiopathology of individual cancers, but also the detailed genetic and epigenetic characteristics, the cancer hallmarks, that clearly show the blueprint of the cancer phenotype is a requisite. Different tumors have a common behavioral pattern, but their specific features at the genetic and epigenetic levels vary to a great extent. Henceforth, with so many failed attempts to therapy, drug formulations and combinations need a focused pre-assessment of the inherent features of individual cancers to destroy the tumors holistically by targeting these features. This review therefore presents innocuous therapeutic regimes by means of CAM and integrative medicine approaches that can specifically target the hallmarks of cancer, using the case of cervical cancer.

## 1. Introduction

For eons, treating cancer using conventional chemo and radiation therapy has been in practice regardless of the many side effects that these two therapies cause. Used separately or combined as concurrent chemoradiation, patients have suffered lethal side effects; and in other instances, the therapy is in fact the cause of morbidity and other lethal outcomes [1,2]. To minimize the occurrence of detrimental side effects and poor treatment outcomes, a team of health professionals including oncologists, physicians, surgeons, nurses and other allied health professionals perform a careful assessment of individual cases to develop the most logical therapeutic option. In the present day, this team of professionals now includes Complementary and Alternative Medicine (CAM) specialists to integrate allopathic medicine with CAM for cancer treatment. This is commonly referred to as integrative medicine and has, in many studies, shown improved post-therapy quality of life than when allopathic medicine is used alone [3].

Publications on the use of natural products for the treatment of disease can be backdated to as early as 1550 BC when the use of therapeutic plants was described in many ancient publications of ancient Egypt, India and China [4]. The increased attention given to CAM therapies now stems back from these ancient discoveries and, to date, there is a promising future in the use of these therapies, especially alongside standard treatments. In addition to CAM and as a part of alternatives to conventional chemo and radiation therapy, modern cancer research has focused on the use of biological therapies to remove tumors with the least risk of side effects. The application of these, requires a deep understanding of not only the etiopathology of cancer but also the genomic and epigenetic characteristics of the cancer to clearly describe its inherent features. Beyond the intrinsic characteristics of the cancer cells, other considerations at the tissue level including the tumor microenvironment and immune environment are important factors in tumor survival and consequently therapeutic outcome. Existing biological and physiological cancer therapies are being considered for integration with CAM and allopathic medicine, with the aim of maximizing tumor eradication while reducing side effects.

In this review, cancer of the uterine cervix (CaCx, cervical cancer) is used as model for the development of such therapies. CaCx is the second most common gynecological malignancy worldwide and recent data, according to the GLOBOCAN estimates, as of 2018 shows that CaCx is the fourth most frequently diagnosed cancer, and also the fourth leading cause of cancer death in women worldwide [5]. CaCx arises from neoplastic transformation of cells at the squamocolumnar junction after initial infection of the basal layer of the cervix by the human papillomavirus (HPV). HPV is well established as the main causative agent of CaCx, reported in nearly all of diagnosed cases [6]. The integration of HPV into the host’s cell genome leads to disruption of the normal cell cycle and immortalization. Since the first description of CaCx by Hippocrates, extensive studies have clarified the mechanisms of growth and maintenance of CaCx, and today CaCx is one of the few cancers whose etiopathology is exceptionally studied and understood. This knowledge has since provided very useful technologies for the prevention, diagnosis and management of the disease.

This review thus presents the usability of CAM and biological therapies used alone and integrated to achieve maximum anticancer effects while minimizing the risk of side effects for conventional chemo and radiation therapies. A full account of the present understanding of HPV and CaCx is given in order to examine the inherent features that play important roles in CaCx and its subsequent response to therapy, which should be the target when designing biological therapies and when considering the use of CAM therapies by means of an integrative medicine approach. However, though this review focuses on CaCx, the dogma and principles of therapies discussed here are inferable to other cancer types by firstly studying the inherent features of each cancer, followed by the assessment of available compounds that can target the respective inherent features. Some features are common between a wide range of cancers, whereas others are cancer specific, hence the need to thoroughly examine features of cancer when designing therapies. For the relevance of this discussion, a brief introduction to the morphology of cancer is given, followed by a detailed etiopathology of CaCx, to set foot on inherent features to be discussed and finally the analysis of those inherent features which CAM and integrative medicine can target to achieve the desired therapeutic outcome proceed.

## 2. The Hallmarks of Cancer

**Figure 1:**
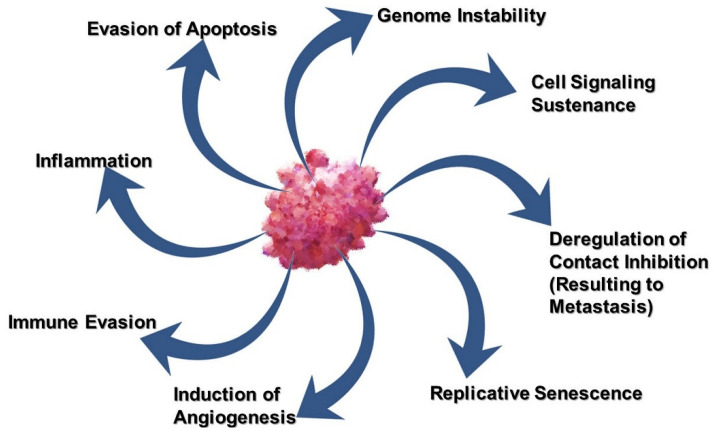
The hallmarks of cancer.

Unlike most diseases that affect the human organism, and can be distinguished from the normal physiological lieu, cancer is a very dubious one that cannot easily be distinguished and yet targeted using medication. In histological examination of tumor tissues, the changes appear more evident but biologically when in vivo, the body’s own cells including the immune system cannot easily identify the cells and pharmacological agents are not any better in achieving the distinction, and this explains the many side effects of cancer therapy. Because of this complexity, present therapies for cancer are being designed to be more specific and directive to the specific characteristics of cancer, referred to as ‘the hallmarks of cancer’. Shown in Figure 1 below are the hallmarks of cancer, characteristics of cancer cells that determine their survival and maintenance in the body.

The annotations in the image above have been established based on the outcomes of extensive research that have shown that genomic alteration in cancer occurs at multiple points, progressively, from a single genetic abnormality to the acquisition of numerous malformations that transform the normal cell to cancer cells [7,8]. Furthermore, tumor cell maintenance has been explained by other colleagues including Otto Warburg, who in 1926 explained the metabolism of cancer cells using aerobic glycolysis [9], a phenomenon commonly referred to as “the Warburg effect”, where energy is acquired form aerobic glycolysis through the increase in the rate of glucose uptake and preferential production of lactate [10,11]. The microenvironment of tumor cells has also been extensively studied [12,13], and shown to be acidic, in which cancer cells tend to thrive better. In CaCx, the integration of the HPV genome into the host’s genetic material is another important hallmark that has therapeutic implications [14]. Specific targeting of these characteristics, therefore, is essential in order to ensure maximum cancer eradication with minimal, if any, effect on normal cells. The following section therefore discusses cervical carcinogenesis to pinpoint the characteristics that can be targeted by CAM and biological therapies.

## 3. Cervical Carcinogenesis

Understanding the features of cancer begins with comprehension of the anatomy and physiology of the tissue of origin. This is very important because the behavior of most cancers, especially in the early stages, is related to the physiology of their tissue of origin. Hence, in the discussion of the inherent features of CaCx, the physiology of the cervix is crucial. The uterine cervix is a cylinder-shaped neck of fibromuscular tissue connecting the vagina and the uterus. The cervix comprises two parts—the part that projects into the vagina, called the portio vaginalis, and a portion that lines the internal part, called the supravaginal portion. Toward the uterus from the cervix, the mucosa lining the cervical canal is known as the endocervix, which separates from the ectocervix at the external os. Though with a continuous surface, the endocervix and ectocervix are microscopically different. The ectocervix is normally covered with non-keratinized stratified squamous epithelial cells and the endocervix is lined with columnar epithelial cells forming a visible drift at the meeting point due to the difference in thickness. Because of this difference, there is a unique interface that is formed where the two epithelia meet, called the squamocolumnar junction [15].

Physiologically, before menarche, the squamocolumnar junction lies on or proximal to the external os where the acid-sensitive columnar cells are not exposed to the low pH of the vagina. However, during puberty and pregnancy, the cervix enlarges and bulges out into the vagina exposing the columnar cells of the endocervix to the acidic environment of the vagina. This phenomenon, referred to as cervical ectopy or ectropion, results in a physiological metaplastic change (i.e., transformation) from columnar to squamous cells. However, the type of metaplasia that occurs at this part of the cervix is not a direct change from columnar into squamous cells, as is the case with most cellular adaptation processes, but rather a replacement of the cells due to proliferation of subcolumnar cuboidal reserve cells, hence called reserve cell hyperplasia or indirect metaplasia. The area where this metaplastic change occurs is called the transformation zone and it is the point at which cervical cells are very susceptible to neoplastic change.

With the physiological events that occur at the transformation zone and the fast cellular turnover of the stratified epithelium of the cervix, HPVs find a safe haven for settlement and continuation of their life cycle. In most women, the activities in the transformation zone result in the development of a mature epithelium composed of normal glycogen-containing metaplastic squamous epithelial cells, very similar to the original squamous cells. With the presence of HPV infections, or sometimes occasionally, it may result in the formation of abnormal, dysplastic epithelium with atypical cells showing impaired structures. These abnormal cells follow either of three outcomes—they may shortly proliferate then spontaneously revert to normal cells, they may continue proliferating to form a dysplastic epithelium or they may slowly progress to become malignant cells usually with persistent HPV infection over a long period [15].

**Figure 2:**
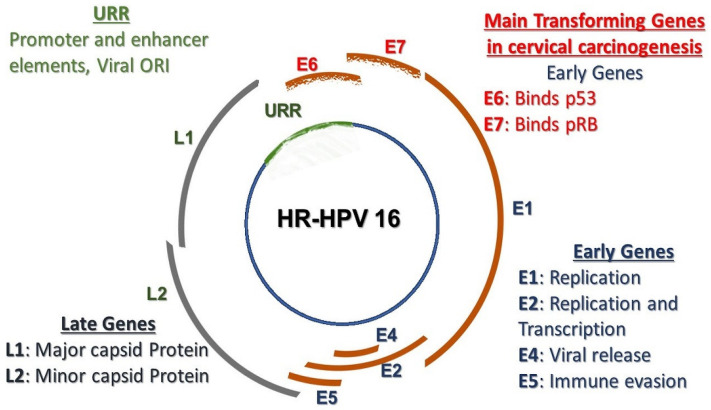
The HPV genome and function of the genes in the viral life cycle.

The advanced knowledge about the structure of the viral particle and full comprehension of the functions of its proteins has significantly contributed so much to the management of CaCx and the development of novel therapeutic methods. All papillomaviruses are small, non-enveloped viral particles containing a single molecule of double-stranded DNA approximately 8000 base pairs in length. Three functional parts can be exclusively denoted in the ORFs. These include the early (E) region, late (L) region, and a long control region (LCR), which contains the non-coding part of the genome but has *cis*-acting elements necessary for replication and transcription of the viral DNA. Viral proteins E1, E2, and E4–E7 are transcribed from the E region and play important roles in viral replication. The L region encodes L1 and L2 proteins that are necessary for assembly of the viral particles [16], as shown in Figure 2 below.

During the development of CaCx, integration events leading to the selective expression of E6 and E7 genes occur. These events then influence the regulation of the cell cycle, mechanisms of apoptosis and cellular proliferation. The E6 and E7 oncoproteins bind to a wide range of host regulatory proteins, specifically p53 tumor suppressor and retinoblastoma proteins, respectively, leading to their degradation and functional inactivation. Furthermore, E7 oncoprotein induces entry of the S phase in cells that are not scheduled for cell division. Direct association and degradation of members of the retinoblastoma (Rb) family that are involved in the regulation of terminal differentiation achieve this. Eventually, this genomic alteration leaves the infected cell unstable and inclined to acquisition of further mutations, which includes several missense, nonsense, silent, non-coding, splice site, non-stop somatic mutations and a few frameshift and in-frame indels [17].

Before the onset of cancer, however, there is an array of noticeable precancerous lesions that have been described as early stages of cancer. Referred to as Cervical Intraepithelial Neoplasia (CIN), these changes indicate the premalignant phase of HPV infection that may regress or progress to cancer. According to the degree of dyskaryosis (extent of damage), CIN is divided into three stages denoted with Roman numerals as grade I, II and III. CIN grade I indicates mild dysplasia and is also referred to as low-grade squamous intraepithelial lesions (LSIL). CIN II and III are moderate and high dysplastic changes, respectively and are known as high-grade squamous intraepithelial lesions (HSIL). CIN III has dysplastic cells covering the entire thickness of the epithelial tissue and is therefore called carcinoma in situ. At this stage, the atypical cells have the highest potential to become invasive and can ultimately invade the basement membrane causing CaCx.

## 4. Innocuous Integrative Oncology for Cervical Cancer

The ultimate goal of therapy is to achieve physiological normalcy post-therapy, but this is not always the case for cancer because of the toxicity of the available allopathic medicine to date. Current conventional treatments approved for CaCx include surgery, chemotherapy, and radiation and have numerous side effects. Because of the characteristics of cancer shown in Figure 1 above, it is evidently not easy to develop a therapy that completely destroys the tumor without affecting normal tissue. To date, the acceptability of approved therapies other than surgery depends on their capacity to reduce cancer growth at doses that have noticeable but with the least side effects. Although the decision for therapy is made having considered many variables including the size and shape of the tumor at diagnosis, the stage of the cancer, the woman’s age and general medical conditions, the individual’s desire to have children later in life, there are still numerous side effects during and after treatment. These have been substantially reviewed elsewhere [18,19,20] and summarized in Table 1 below.

### 4.1. Complementary and Alternative Medicine (CAM) Therapies

Complementary and Alternative Medicine (CAM) Therapy is simply defined as a group of all medical and health care systems, practices and products that are not normally considered to be conventional medicine. The two are technically the same thing, only defined as ‘complementary’ when they are used together with conventional medicine and ‘alternative’ when they are used in place of conventional medicine [21]. This definition denotes a very broad part of medicine that includes mind–body interventions, manipulative and body-based procedures, energy therapies, biologically-based treatments, and alternative medical systems. The common use of complementary therapies in oncology is not necessary with the attempt to cure but rather as supportive therapies and hence mainly used to alleviate symptoms of conventional therapy and also in palliative care. Shown in Table 2 below are common CAM therapies used as part of cancer therapy. 

However, there are many anticancer properties in many different forms of CAM especially most nutraceuticals including vitamins, herbs and supplements [36] details of which are given in proceeding sections below. However, these need very careful administration when used alongside allopathic medicine, which is the case when treating cancer. When used alongside chemo agents, certain compounds in these natural products can affect how the drug works [37]. Additionally, cancer is a complex disease with many molecular processes that are responsible for the tumor phenotype and different compounds can slow down or speed up the cancer growth. Hence, a careful assessment is utterly important. In all conscience, for complex diseases such as cancer, even allopathic therapies to date have many limitations in eradicating the tumor. Likewise CAM therapies cannot be used alone to treat cancer. Therefore, integrative medicine is the common practice in the modern day, where CAM therapies are used alongside allopathic medicine to treat cancer. The correct use of integrative medicine enables a holistic approach to therapy that ensures that the body’s innate healing response works together with medical interventions to effect on not only the disease but also the other aspect of the body, e.g., mind, spirit and overall health, things that do have so much influence on the outcome of mainstream therapies [38].

### 4.2. Biological Therapies

In addition to the benefits of natural products and compounds, less invasive biological interventions, are useful for treating cancer. Conventional chemotherapy, radiotherapy and surgical therapy are commonly used for cancer, but this age has seen a great advancement in studies performed on non-conventional medical treatments with many of those being investigated in thousands of clinical trials at any given time. More research in vitro also continuously presents the basis of novel approaches to therapy and elaborates on cancer cell biology and their response to several therapeutic agents. To minimize the risk of side effects induced by synthetic pharmaceutics and invasive therapies, biological therapies as an alternative approach for treating cancer are the modern therapeutic options. Most of these, though used frequently in many clinical trials, have not been approved as allopathic (mainstream) therapies, hence they are included in the category of alternative medicine. Biologically based therapy for cancer involves the use of living organisms, substances derived from living organisms, or laboratory-produced versions of such substances to treat the disease. Their application stops cancer by directly acting against cancers or indirectly through stimulation of the body’s immune system to act against the cancer cells. Another class of biological therapies also stops cancer growth by interfering with molecules that play a maintenance role for tumor growth and progression [39,40].

Very few biological therapies have since been approved (see FDA-approved biological therapies), and many others are still in clinical trials and in vitro. Overall, biological therapies are a focus of modern cancer therapeutics. Examples of such therapies include but are not limited to immune checkpoint modulators, drug immune conjugates, targeted drug therapy, direct monoclonal antibodies (mAbs), oncolytic virus (OV) therapy, angiogenesis inhibitors, cancer vaccines, chimeric antigen receptor (CAR) T cell therapy, gene therapy, and cytokine therapy. Because biological therapies are recent discoveries with so much work continuously being performed, there are certain limitations in their nomenclature. Thus, other oncologists propose the use of another parameter that describes the mode of action of the therapy in terms of whether it involves a physiological process. Physiological therapies therefore refer to those that work by means of a function of the human body. Furthermore, other scientists will refer to biological therapies as immuno-oncology therapies, and distinction is lacking between certain categories, resulting in the inclusion of a particular type in more than one category in as far as classification in concerned. For the purpose of this discussion, therefore, the common biological therapies are classified in relevance to the discourse that follows in the discussion of inherent features of CaCx that these therapies work on.

#### 4.2.1. Immunotherapies

Molecular complementarity between cell surface antigens and their corresponding immune elements, i.e., antibodies and cell recognition sites of lymphocytes, is the basis of immune action. Because of this mode of action, cells of the body need a mechanism of avoiding immune destruction even during an immune reaction. Apart from the Major Histocompatibility Complexes (MHCs) that the immune cells recognize to avoid destroying self-cells, an additional regulatory mechanism during an immune reaction to pathogens in the body occurs to ensure self-tolerance [41]. Immune checkpoints are therefore conveyed and represented by two different forms of signals including (1) co-stimulatory immune checkpoints, those that stimulate the progression of immune reactions, and (2) co-inhibitory immune checkpoints, those that inhibit the progression of immune reactions. Among the co-inhibitory immune checkpoints are cytotoxic T-lymphocyte-associated antigen 4 (CTLA-4), programmed cell death protein 1 (PD1), V-domain Ig suppressor of T cell activation (VISTA), lymphocyte-activation gene (LAG)-3 [42,43].

**Figure 3:**
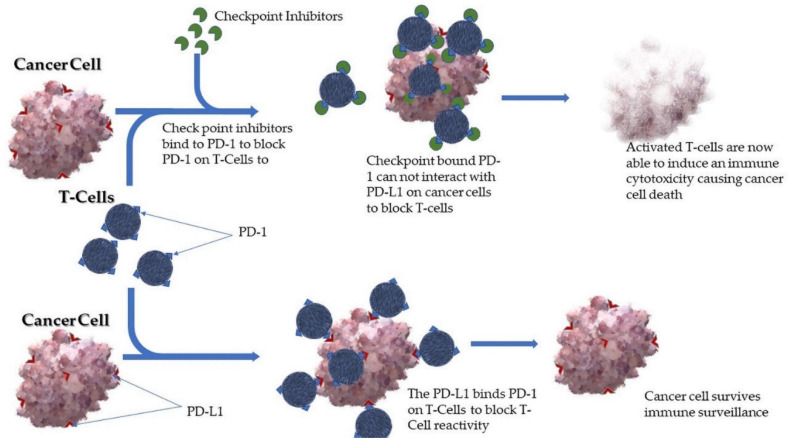
Schematic representation of an immune checkpoint inhibitor.

The aforementioned checkpoints are designed to protect normal body cells from immune damage during the immune reaction to the pathogens. Ideally, cancer cells should be recognized by the immune system as foreign cells because of the many genetic and epigenetic changes that occur during tumorigenesis, which provide antigens referred to as tumor-associated antigens (TAAs). These TAAs initially are recognized by the immune system and without a mechanism by the cancer cell to resist the immune action, tumors would not survive in the body. Therefore, during the development of the tumor, the cancer cells shrewdly dysregulate these immune checkpoints to escape immune attack even in the presence of TAAs and immune surveillance [44]. The cancer cells also change their antigen structures, downregulate important MHC molecules and/or secrete substance that suppress the action of the immune system. Most cancer cells including cervical, upregulate PD1 ligands which target the PD-1 on T cells, thereby suppressing the antitumor actions of T cells (Figure 3). Because of this observed phenomenon, immunotherapies have over the years been a commonly used biological therapy. The application of immunotherapies is therefore based on the principle that blocking or modulating the ligands and receptors that are necessary for the interaction of the cancer cell and immune cell for immune tolerance does prevent the cancer cell from evading the immune reaction. The immune checkpoints are initiated by ligand–receptor interactions that can be blocked using mAbs, or recombinant technology.

#### 4.2.2. Oncolytic Virus (OV) Therapy

Another interesting immune system activator is the OV therapy, which uses replication-competent viruses to destroy cancer cells. The principle behind OV therapy is that, OVs bypass normal cells to infect tumors resulting in continuous replication inside the cancer cells thereby injuring the entire tumor [45]. The OVs are genetically modified to achieve tumor selectivity plus oncolysis. Over the years, different types of OVs have been modified from viruses including the common Retroviruses, Paramyxoviruses, Adenoviruses and Herpes simplex viruses (HSV). With a tiny minority of government-approved OVs including the talimogene laherparepvec (T-VEC), adenovirus, and an HSV, most OVs are still on the road to approval, currently in clinical trials and in vitro [46].

Though very effective, as demonstrated by the efficacy of the approved OVs, data regarding OV therapy suggest the need for further scientific expositions as there are a few limitations thus far. Like most therapies, a very important observation with the application of OVs is the need for a carefully performed optimization protocol to find the balance between maximal antitumor immunity and minimal antiviral immunity. This observation is a typical example of a hormetic-type response of living cells to stressful substance. Hormesis is a phenomenon where processes in a cell would display a biphasic response after exposure to increasing amounts of a substance or condition, and is characterized by a low-dose response that is opposite in effect to that seen at high doses, i.e., stimulation at low doses versus inhibition at a higher dose [47]. In the presence of such a response, the hormetic zone would refer to the range where there is an favorable biological response to low exposures with no effect at high doses. This means that the OVs injected in the body should not exceed the dose of the hormetic zone and that requires enough information about the tumor including tumor mass, site and features of its microenvironment.

#### 4.2.3. Anticancer Vaccines

Apart from the well-known and highly recommended HPV vaccines as prophylactic for CaCx, vaccination for treatment is another growing field in cancer management. The HPV vaccines, Cervarix and Gardasil, are given to new born or adolescent girls to prevent cervical carcinogenesis after exposure to HPV in their life time. For diagnosed cases, a therapeutic vaccine can also be designed for curative purposes and are in their application also categorized as immunotherapies. When using therapeutic vaccines, the idea is based on the concept of using overexpressed tumor-associated carbohydrate antigens (TACAs), proteins and enzymes that are antigenic in cancer cells [48]. A common example is the sipuleucel-T vaccine for the prostatic acid phosphatase (PAP) in prostate cancer, formerly approved by the American FDA [49,50]. In the case of virally induced cancer including CaCx, viral proteins and oncoproteins can also be used as targets for therapeutic vaccines. However, hormesis as described previous is also an issue with the use of anticancer vaccines.

#### 4.2.4. Targeted Therapies

The search for agents that can be directed towards cancer cells to avoid normal tissue toxicity led to the discovery of varying types of targeted therapies. Simplified, a targeted therapy is a type of treatment that uses drugs or other substances to identify and attack specific cancer cells without harming normal cells [51]. Certain immunological agents are also categorized as targeted therapies. A common targeted therapy that uses mAbs produced from a single clone of B lymphocyte, also called monoclonal antibody therapy, employs the principle of specific Ab binding to specific antigens and hence achieving directed/targeted tumor destruction specific enough to reduce severe side effects [52]. Because of the unique antigen–Ab interactions, the Abs can only recognize and bind to the cancer cell that has the corresponding antigen, leading to antitumor reactions by either blocking cell growth, preventing metastasis or inducing immunological reactions that cause cytotoxicity. These mAbs can be used alone due to their immunological nature, or can be used as carrier molecules for antitumor drugs, toxins, or radioactive materials directly to cancer cells [53].

**Figure 4:**
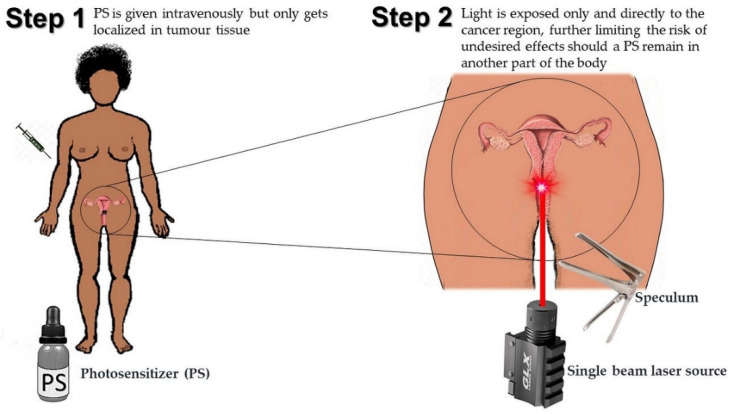
The double selectivity and specificity of Photodynamic Therapy (PDT).

Another important type of targeted therapies that can also activate the immune system at the tumor site but also has more prominent primary cytotoxic effects in itself is Photodynamic Therapy (PDT). Currently approved in many countries and by the American FDA, for treatment of some solid tumors, PDT has emerged as a potential treatment modality for CIN and CaCx and shows many advantages over most putative CaCx therapies [53,54]. Pharmacologically, PDT uses not one, but two principles of selectivity and specificity to maximally target the tumor cells with no effect on distant organs and very minimal or rather negligible effects on surrounding cells [55,56]. This phenomenon is so because PDT uses a couple of individual substances that are dependent on each other to attain reactivity, and these independent substance are administered separately. In the first step, a photosensitizing compound (photosensitizer or PS) is administered intravenously, intramuscularly or topically and given time to selectively localized in the tumor because of the tumors affinity to the drug. Once the drug has localized in the tumor, the second step involves light irradiation, usually using a laser, specifically directed at the tumor in a single beam. The light then activates the internalized drug and in the presence of tissue oxygen, cytotoxic oxygen species and radicals are formed, which destroy the tumor cells from within, and also from external effects upon destruction of the vasculature and activation of the immune system [54,55,56]. The way PDT is delivered gives it not just one but two levels of specificity, where the PS is selectively localized in the tumor tissue, and light is only directed to the cancer region as shown in Figure 4 below.

## 5. Cancer Hallmarks Targetable by Innocuous Integrative Oncology

The aforementioned hallmarks of cancer in Section 2 are necessary for cancer maintenance and survival. Attacking these features using innocuous biological therapies and CAM not only maximizes therapeutic efficacy, but also minimizes the risk of side effects since these features are either not found on normal tissues, or are expressed differently. These features therefore provide many opportunities for designing therapies that have the highest potential in eradicating tumor cells. The section below reviews how these features can be targeted in CaCx.

### 5.1. Targeting the Genomic Instability

Although they mimic normal cells of the body phenotypically, cancer cells have genotype alterations that are better suited to be pharmacological agents than the phenotypic properties currently used as targets for most therapies including chemo and radiation. These genomic alterations give the cancer cells inherent features for their proliferation and maintenance and are unique features that can be used to distinguish them from their normal cell counterparts. The integration of the HPV genome into the cells’ own genome initiates a cascade of genetic instabilities that result first in the impairment of the p53 gene and the members of RB gene with a consequent increase in acquisition of more gene alterations and somatic derangements. In all cancers including CaCx, these genetic abnormalities allow for evasion of cell cycle control pathways and the initiation of the accumulation of somatic mutations [57,58]. This mechanism is the common initiation step for most cancers and is a significant tool that has therapeutic implications.

However, in the midst of numerous genetic derangements that lead to cancer, a more important tool is the determination of mutational significance in cancer (MuSiC), which is aimed at separating the significant genetic events which are the important drivers in the pathogenesis of the cancer from the other less significant mutations that accompany the process [59]. During cervical tumorigenesis, many specific gene alterations directly related to the initial integration of HPV viral genome lead to a number of significantly mutated genes (SMGs) that provide data that are useful for designing gene-targeted therapies and immunotherapies. Most commonly affected genes in CaCx include HLA-A, Transforming Growth Factor Receptor 2 (TGFBR2), SHK-Binding Protein 1 (SHKBP1), Erb-B2 Receptor Tyrosine Kinase 3 (ERBB3), Caspase 8 (CASP8). These have prominent opportunities for therapy, for example the ERBB3 (HER3), which can be targeted using mAbs [60]. 

Genome editing and gene therapies have shifted the therapeutic perspective by focusing on treating disease at the genetic level with the use of nucleic acids. Gene therapies have previously been discussed for the treatment of a wide range of different cancers [61,62,63]. However, many issues regarding their applications, pharmacology and ethical implications have been debated for decades [64]. The idea of gene therapies is to use a method that directly affects the function of a gene in order to archive a therapeutic target. Different ways of approaching gene therapy have been described especially those that use a viral vector [65], or nanoparticles [66] to effect cancer causing genes, e.g., p53, or to effect on immune T cells that boosts immune reaction against cancer also called immunogenic therapy. The constitutive role of HPV and its direct association with p53 and members of the RB gene suggests the use of gene therapies [67]. Because of the restricted access to genetic material in the body and the fact that most cancers are noted during the progression and advanced stages, targeting the genetic material can be somewhat problematic unless in cases where screening results in the confirmation of mutations at the initiation stage before acquisition of alterations in SMGs. However, these mutations lead to the impairment of molecular activities of the cell, the stage at which other therapeutic regimes can have much effect, as describe in the following section.

### 5.2. Targeting Cell Signaling

Genetic alterations are very closely correlated with the alteration in their corresponding molecular pathways. Hence, another important hallmark of cancer is the alteration of cell signaling that enables the cancer cells to grow and create a sustainable environment for themselves. At the molecular level, numerous therapeutic regimes can be developed from biological therapies and CAM therapies, including nutraceuticals, nanoparticles (Ns), and synthetic drugs. Although most nutraceuticals are evidenced for prevention and management of cancer, their mode of action can also be utilized for treatment purposes as part of an integrative treatment regime. Naturally occurring nutraceuticals are easy to use in certain instances—there is a need to incorporate or add nutraceuticals to food and this has a significant impact on the physicochemical and sensory characteristics and structures of the original food, a variable that needs proper assessment [24].

Cell signaling comprises a larger aspect of molecular mechanisms in the pathology of cancer. Pathological events leading to tumorigeneses and tumor maintenance in most cancers including CaCx involve the activation of Wnt/b-catenin, phosphatidylinositol-3 kinase (PI3K)/AKT, epidermal grow factor receptor (EGFR)/vascular endothelial growth factor receptor (VEGFR), Ras/Raf/MEK/ERK, and the deactivation of cellular apoptotic pathways. Although varying phenotypes of CaCx exist in different individuals, one or more of the alterations in these pathways are found in the majority of cases, carried on throughout the course of the disease, and provide an opportunity for the implementation of CAM and design of biological therapies that target cell signaling.

#### 5.2.1. Wnt Signaling Pathway

The Wnt signaling pathway plays a major role in the tumorigenic transformation of HPV-infected cervical cells [65]. Activation of the Wnt signaling pathway leads to a series of events that culminate to the accumulation of b-catenin in the cytoplasm, which promotes its entrancement to the nucleus, finally activating the transcription of c-myc and cyclin D. Although it is not feasible to target the Wnt/b-catenin pathway using a single forward approach due to its complexity and the varying associated conduits within the pathway, numerous compounds with significant effects on the pathway are available. Naturally occurring polyphenols attenuate Wnt/b-catenin signaling via the induction of cellular degradation of the b-catenin alongside a reduction in the nuclear accumulation of b-catenin. The advantage of polyphenols is that they are abundant in nature, are found in a variety of foods and can be extracted and used with less alteration. These compounds have both defensive and therapeutic properties against most tumors. In CaCx, polyphenols have been qualified for the sensitization of cancer cells to chemo and radiation [23]. Polyphenols are categorized using five separate subgroups namely phenolic acids, curcuminoids flavonoids, stilbenes, and lignans. Specific mAbs and peptides directed against different parts of the Wnt/b-catenin pathway can be used [66]. For the purpose of maximally effecting on this pathway therefore, an integrative approach using nutraceutical containing polyphenols and mAb therapy can potentially produce better outcomes. To date, there are no reported cases of a possible antagonistic mechanism between natural polyphenols and mAbs.

#### 5.2.2. EGFR/VEGF Pathway

An important member of the tyrosine kinase receptor family, EGFR with its family members Her/Erb, and ligands, are important regulators of carcinogenesis in many cancers including cervical. In addition to its varying downstream pathways, an important downstream activity of EGFR is induction of the synthesis of VEGF upon stimulation by EGF-activated signals, forming the EGFR/VEGF pathway. VEGF in turn, is an angiogenic factor, a dimeric glycoprotein that plays fundamental roles in the neogenesis of vascular (and lymphatic) channels, both in physiological developmental processes and pathological conditions such as cancer [68]. Because of its role in neoangiogenesis, this pathway is vital for tumor maintenance and metastasis. Opportunely, there are numerous EGFR/VEGF blockers that effectively target components of this pathway in use, in clinical trials and in vitro. Bevacizumab is an FDA-approved CaCx targeted therapeutic agent for the treatment of advanced/metastic CaCx, and recurrent disease [69,70]. This drug is a recombinant humanized mAb, that immunologically targets VEGF-A in the EGFR/VEGF pathway. Bevacizumab has been used for CaCx treatment in many clinical trials that led to its approval, and continues to be used, with good patient prognosis despite its high toxicity levels in some cases according to expert opinion. A tyrosine kinase inhibitor (TKI), pazopanib also directly affects VEGF and is currently used for renal cell carcinoma and other soft-tissue tumors though not currently approved for CaCx [71,72].

Many other anti-EGFR mAbs and TKIs that target EGF and EGFR are available and widely used for treating other cancers. Lapatinib, a potent TKI commonly used in the treatment of HER2-positive breast cancer is a synthetic drug that targets both EGFR and another member is the EGFR family ErbB2 (HER2). Though not widely used for CaCx, clinical trials using lapatinib show potential use of this drug for the treatment of CaCx [72]. Other TIKs at varying stages of investigation include imatinib, Erlotinib, Matuzumab and Gefitinib. Nimotuzumab is a mAb that shows very significant anti-EGFR activity and has been studied in many CaCx clinical trials, with promising results in limiting the progression of cancer [73]. A similar drug in action, Cetuximab, a chimeric mAb that blocks this pathway by binding to the receptor causing it dimerization and downregulation, also has promising anticancer effects [74].

#### 5.2.3. PI3K/Akt Signaling Pathway

The PI3K/Akt signaling pathway is a very important element for the enhancement of cancer cell proliferation and their survival in many solid tumors including CaCx. The PI3K/Akt signaling pathway exerts its effects by downregulating the role of the Ras signaling in the cell cycle, and modification of receptor signal transduction via its kinase activity. This pathway is also one of the many downstream EGFR-associated pathways described above. Blocking the pathway using PI3K inhibitors is a considerable method for weakening early stage CaCx. Many compounds that directly interfere with the catalytic subunit of PI3K by competing with ATP binding are available to avoid propagation of CaCx. A naturally occurring metabolite of *Penicillium funiculosum* called Wortmannin (Wm), which, apart from its direct effect on PI3K pathway, possesses features of chemosensitization and radiosensitization is available for CaCx [75].

Another compound with similar properties, a derivative from flavonoid quercetin, 2-(4-morpholinyl)-8-phenyl-chromone (LY294002) also targets PI3K [76]. However, unlike Wm, LY294002 possesses a very short life span with a higher off-target activity and high toxicity, which restricts its clinical application. Several naturally occurring compounds have anti-PI3K/Akt activity. Common ones include, genistein found in Soy foods, oridonin from *Rabdosia rubescens*, triptolide from *Tripterygium wilfordii*, quercetin and rapamycin produced from *Streptomyces hygroscopicus*, which targets and inhibits a downstream component of the PI3K/Akt pathway known as the mammalian target of rapamycin (mTOR) [77,78].

#### 5.2.4. Ras/Raf/MEK/ERK Signaling Pathway

Another important downstream EGFR-associated pathway is the Ras/Raf/MEK/ERK signaling pathway, which plays vital roles in normal cell physiology. However, similar to most of these signaling pathways, the Ras/Raf/MEK/ERK signaling pathway can be activated in most cancers including CaCx [79]. EGF also plays an important role in the activation of this pathway, leading to tumorigenesis and tumor maintenance. Briefly, EGF-associated complexes involving RAF lead to the induction of MEK1 and MEK2, which in turn phosphorylate and activate ERK1 and ERK2. Eventually, ERK1/2 acts on a vast number of cell regulator substrates in both the nucleus and cytoplasm, including cell cycle kinases, transcription factors and gene expression. In addition, Raf family members are upstream activators of the ERK, which, when activated, induce the signaling of ERK1/2 [80]. With so many intermediate signal regulators in Ras/Raf/MEK/ERK signaling comes numerous inhibitor molecules that can be directed at different stages of this pathway. Highly specific MEK1/2 inhibitors are under review including ERK1/2 antisense oligonucleotides, PD98059 and U0126 [81]. The first inhibits ERK1/2 protein expression and reduces its concentration in the cell and the other two inhibitors suppress ERK1/2 phosphorylation without changing the concentration of ERK1/2 in the cell. Because Raf is an important upstream regulator, drugs that inhibit Raf, e.g., BAY43-9006 and Sorafenib, are being considered for treating CaCx. Importantly, since the EGF is an important regulator for this pathway, drugs that inhibit EGFR are similarly useful in preventing activation of the Raf/MEK/ERK pathway, which includes the possible incorporation of the nutraceuticals previously described.

In principle, most of these pathways are related in one way or the other, with the activation of one pathway imitating the regulation of another. Because of this phenomenon, targeting one pathway, especially those upstream, results in the impairment of several other pathways and, in all practical senses, the combination of drugs that inhibit different pathways and different phases of their signaling has potential for resolute attenuation of cancer propagation. However, care should be taken in the dosing and delivery of such drugs and compounds due to the presence of similar pathways in normal cells that are essential for continuation of life. In addition to that, the pharmacological interaction between the different compounds when administered together should be evaluated.

### 5.3. Targeting Immune Evasion

Cancer cells have cellular regulation mechanisms for their maintenance and survival in the body. These mechanisms including surface antigen expression (i.e., the TAAs), extracellular vesicles that are secreted to mediate cell-to-cell communication and other neoantigens [44]. At present, mAbs against CTLA4 are used for treatment of certain cancers in most countries and blockers of PD1 are very close to being approved because of the many clinical trials that have shown promising results. As shown previously, PD-L1 molecules on the cancer cells stop the T cell from killing the cancer cell by attaching to the PD-1, a protein on the surface of T cells [82]. With immune checkpoint inhibitors, ligands to prevent the action on PD-L1 are used to activate T cells by blocking the PD-1 on the surface of the T cells. A novel PD-1 checkpoint blocker used for most cancers including CaCx, called Pembrolizumab, has shown promising results for treatment of recurrent tumors [83,84]. While nutraceuticals and mAbs inhibit angiogenesis at the molecular level, PDT can cause damage to intact blood vessels via its cytotoxicity. Targeted therapies including PDT and mAb therapy have also been useful in effecting cytotoxicity and tissue damage.

Although there are currently no licensed therapeutic HPV vaccines for cervical cancer, studies have validated that vaccines have both prophylactic and therapeutic effects on cervical cancer. Che et al. 2020 [85] studied the HPV16 E7 43-77 peptide and the adjuvant unmethylated cytos 7ine-phosphate-guanosine oligodeoxynucleotide and demonstrated that this vaccine had substantial effects on systemic immune responses and the tumor microenvironment (TME) in a mouse model of cervical cancer. Their results indicated a significant increase in interferon (IFN)-γ-producing CD4 and CD8 T cells and tumor-infiltrating CD4 and CD8 T cells. Additionally, there was a marked decrease in splenic myeloid-derived suppressor cells (MDSCs), regulatory T cells (Tregs), and M2-polarized tumor-associated macrophages.

### 5.4. Targeting Angiogenesis

In some studies, mAbs, as described previously, can be used in a combination of two or more mAbs to achieve a synergistic antiangiogenesis approach, which has shown better outcomes [86,87]. In most cases, angiogenesis inhibitors have also been used alongside conventional chemo and radiation therapies. In addition to these combinations, inhibition synergism can also be achieved by the integration of a mAb and certain nutraceuticals. Such compounds include but are not limited to the polyphenols previously described, curcumin and epigallocatechin-3-gallate from green tea [88]. The integrative medicine approach using angiogenesis inhibitors and nutraceuticals has previously been examined [89]. Although there is limited understanding of the pharmacological interactions between these two regimes, their combination for a possible synergistic action is pertinent.

Bevacizumab, previously described above, is the most common mAb therapy and approved in most countries. In addition to Bevacizumab, other mAbs can be used to target surface and intracellular proteins of the cancer and cause cytostatic or cytocidal effects within the cell. A personalized neoantigen vaccine can also be applied. The added advantage of the use of neoantigens is that they are new-fangled antigens produced mainly due to the infection, i.e., neo for new, and hence are only found on growing cancer cells [90]. This technique has the most potential for the least side effects, if any, since the vaccine can only induce an immune response specifically targeting the mutated cancer cells without cross effects on normal cells unless there is a secondary impact.

### 5.5. Targeting Oncoproteins

The role of HPV in cervical carcinogenesis makes the HPV oncoproteins good candidates for therapeutic targeting. Although there is a minority of HPV-negative CaCx, up to 98% of CaCxs are HPV positive and the important function that these proteins play in the development and maintenance of CaCx provides an opportunity through which therapies can be designed. Because of the important role that HPV oncoproteins play, hr-HPV E6 and E7 oncoproteins make attractive therapeutic targets [91,92]. The E6 and E7 oncoproteins have been studied substantially and have been shown to localize inside the cell in high concentrations, the site and concentration of which depends on many factors including the stage of cell cycle and cell confluence in vitro. The presence of the E6 and E7 oncoproteins in the nucleus, perinuclear region, cytoplasm and cell membrane, provides an opportunity for targeting using mAbs, therapeutic vaccines and nutraceutical described previous [93].

Although there are no approved oncoprotein-targeting drugs to date, many studies both in vitro and in vivo have indicated the potential of these drugs. Additionally, anticancer vaccines against HPV oncoprotiens and p53 are being explored. However, Targeting HPV oncoproteins is limited by the stage of cancer. Although the proteins are expressed in cancerous cells during the course of disease, cancers acquire numerous other features that are necessary for cancer propagation. That is to say, HPV is necessary for carcinogenesis, but after the initial integration of the HPV genome that initiates transformation, numerous other processes that result in genetic instability and the acquisition of other mutations occur, resulting in genetically mutated phenotypes that become cancer cell independent of the oncoprotein’s transforming effect for tumor maintenance. This is the reason why the approach is more rational for CIN and early stage disease. However, the presence of these proteins still provides an opportunity for targeted drug delivery of chemotherapeutic agents and other anticancer drugs.

### 5.6. Targeting the Tumor Microenvironment

In addition to these, the tumor microenvironment contains elements including the extracellular matrix, blood vasculature, immune cells and fibroblasts that play important roles in the survival of the cancer cells. All these have therapeutic implications and many biological therapies at varying stages of investigations have been designed. A minority of those have since been approved (see FDA-approved biological therapies), and many others are still in clinical trials and in vitro. tumor-associated macrophages, tumor-associated blood vessels (TABVs), tumor-associated stromal cells (TASCs), and the acidic surrounding in the tumor extracellular matrices are very important regulators of the tumor microenvironment. The tumor microenvironment is an important hallmark of cancers, hence a prefect target for safer therapies. In the tumor microenvironment, macrophages play a very important role in carcinogenesis [94].

Another important feature in the tumor microenvironment is the presence of CD4+ T regulatory cells (Tregs). A small number of Tregs circulate in the body naturally to function as mediators of immune suppression to prevent autoimmune diseases. However, in cancer, Tregs are recruited by tumor cells into the tumor microenvironment in order to inhibit antitumor immunity [95]. Visser et al. [96] examined the frequencies, phenotype and activity of (Tregs) in patients with cervical neoplasia and showed that both CIN and cervical cancer patients had increased Tregs in the peripheral blood. In the tumor microenvironment, these cells interfere with the action of the immune system by suppressing antigen-presenting cells (APCs) through depletion of immune-stimulating cytokines followed by the expression of CTLA-4, which inhibits the APCs [97]. Additionally, they also limit the efficiency of cancer immunotherapy. Using anti-CTLA-4 antibodies including ipilimumab (FDA approved for treatment of advanced melanoma) effectively reduces the action and number of Tregs in the blood, with subsequent activation of cytotoxic T cells [98]. Although there are no approved anti-CTLA-4-Treg inhibitors for CaCx to date, the presence of Tregs in HPV-induced cancers including CaCx, suggests the use of monoclonal antibodies to act as check point inhibitors in CaCx, but this remains to be an area of research [99,100]. Ji et al investigated the combination of radiotherapy and suppression of Tregs in rectal cancer and observed that this dual approach enhanced the efficacy of therapy with inhibition of metastasis [101].

### 5.7. Therapeutic Strategies for Cervical Cancer to Date

At present, numerous therapeutic strategies for cervical cancer are available in clinical trials, in vitro studies and in clinical practice. Current approved treatments for cervical cancer include chemotherapy, radiation, surgery, immunotherapy and targeted therapy. Conization is the removal of a cone-shaped piece of tissue from the cervix and cervical canal and is the most widely used treatment for CINs, carcinoma in situ and stage I tumors in most countries. The tissue can be removed using a scalpel (cold-knife conization); using an electrical current passed through a thin wire loop as a knife called loop electrosurgical excision procedure (LEEP); or using a laser beam to cut the tissue. Radical hysterectomy or, most times due to technicalities, simple hysterectomy are often recommended as a cure for locally advanced cervical cancer. Other surgical procedures in use include pelvic exenteration (removal of all organs of the pelvis), lymphadenectomy (surgical removal of the lymph nodes), and radical trachelectomy (the removal of the cervix and its surrounding tissue, leaving the body of the uterus). Women with early stage disease who wish to maintain fertility opt for radical trachelectomy with lymphadenectomy. 

Chemotherapeutic drugs are mainly used as neoadjuvant or adjuvant therapies for cervical cancer. Drugs used in many countries include 5-fluorouracil, paclitaxel, cy-clophosphamide, cisplatin, carboplatin, and ifosfamide. In advanced localized cases, concurrent/concomitant chemoradiation therapy (CCRT) is used as treatment or for palliative care. Because of the many side effects of external radiation on nearby organs such as the bowel and bladder, internal radiotherapy (brachytherapy) is preferable. After metastasis, Bevacizumab is used as a targeted therapy for prevention of angiogenesis by binding to vascular endothelial growth factor (VEGF). Bevacizumab is also the treatment of option for recurrent cancer. Thus, the only approved innocuous therapies widely accepted for CaCx mAbs for targeted therapy, and some PD-L1 immune checkpoint inhibitors [102,103]. 

## 6. Conclusions

In conclusion, here we described and evaluated the use of CAM therapies, non-invasive biological therapies and their combinations in the form of integrative medicine for treating CaCx. With most, if not all, types of cancer, this approach can be considered in order to maximize the benefits of therapies singularly or combined. However, there is still a longer route to the acceptability and use of the varying types of therapies due to ethical reasons and the need for clinical trials before their widespread acceptance and approval. Cellular changes that occur to the cancer cell at the genome (i.e., DNA), molecular, and morphological levels distinguish CaCx cells from their normal surrounding tissue and also from other cancer cells for all practical applications. While allopathic medicine focuses on the tumor with a specific mode of action, integrative oncology regimes can be designed to focus on a wide range of targets in the cancer tissue. Additionally, we have analyzed the inherent features of cervical cancer that can be used as targets for many nutraceuticals, biological therapies and other CAM therapies. This case of cervical cancer can be applied to the broad spectrum of cancers, having examined their inherent features at the genetic, molecular, cellular, tissue and organism levels.

## Figures and Tables

**Table 1:** Common side effects of conventional therapy to cervical cancer.

Chemo and Radiation	Surgery
Anemia Appetite loss Constipation Delirium Diarrhea Edema (swelling) Fatigue Hair loss (alopecia) Nausea and vomiting Pain Sleep problems and insomnia	Fertility loss Physical and psychological sexual disorientation Pain at the incision site Blood loss Infection Damage to the bladder or intestines

**Table 2:** Complementary and Alternative Medicine (CAM) Therapies.

Therapy	References
Nutraceuticals	[22,23,24]
Acupuncture	[25,26,27]
Massage therapy	[28]
Music therapy	[29,30,31]
Aromatherapy	[31]
Hypnosis	[32,33]
Meditation	[34]
Yoga	[35]

## Data Availability

Not applicable.
